# Remarks in Relation to the Pathological Anatomy of the Mucous Membrane of the Intestinal Canal during Infancy

**Published:** 1848-02

**Authors:** 

**Affiliations:** Frankfort on the Main; Frankfort on the Main


### ARTICLE IV.

*Remarks in relation to the Pathological Anatomy of the mucous membrane of the intestinal canal during infancy.* By Drs. Friedleben and Flesch, of Frankfort on the Main. From Henle's and Professor Lietschrift's v. 3, 1846—1847. Translated from the German, for this Journal, by Daniel Stahl, M. D., of Quincy, Illinois.

*Normal condition of the intestinal mucous membrane in the first period of life.*—It is constantly covered with a *thin layer*

(from the fecal matters,) of whitish or yellowish, mucous; a somewhat thicker layer leads us to suspect a morbid change. The mucous suffers no change of colour, from the morbid changes of the tissures, (except accidently from medicine, &c., &c.) This layer of mucus, if it is very thin, and the parieties of the intestines atrophic and anæmic, can easily be taken for the mucous membrane itself. The *colour* of the mucous membrane is, in its natural condition, sometimes yellowish, at others grayish-white; changes can take place by imbibition of bile, medicines, blood, and by putrifaction. The *folds* in the small and large intestinal canal are present, even in the youngest subjects. The *adhesion* of the mucous membrane with the other membranes is very strong, excepting in the colon, where it is a little loose. The *consistence* is always very firm, so that the mucous membrane can never be removed by scraping with the back of a knife. The *solitar-glands* (isolated glands, I suppose—transl.) of the small intestines are not, in a normal condition, visible; neither are those of the large intestines in the natural condition; yet small, roundish, opaque points, which, however, are not prominent, are frequently seen. *Lieberkuehn's glands* cannot be seen with the naked eye, except in rare instances, in the form of small depressions. *Peyer's patches* are, even in early life, visible; their number changes from thirty-six, to sixteen, and even to six; the largest are near the valve of the coecum; they are smaller towards the upper part. Their form is eliptical, (from 4‴ to 1½″ length, and 2″ to 4‴ breadth.) Those on the ilium are well circumscribed. These glandular patches, can in the healthy subject, only be seen (with the exception of those near the valve,) by holding the gut against the light; they will then show themselves as opaque spots. Their easy visibility is a sign that they are morbidly affected. Their colour is in a healthy condition, either not at all, or only by a slightly grey colouring, distinguished from that of the mucous membrane; there are never to be seen vascular arborisations upon them. The arcolar structure of these glands is very early present.

The pathological condition of the intestinal mucous membrane of infants, (children in the first year,) in *atrophy* and *diarrhœa*, is according to the author's observations, the following:

A. CONGESTION.

*A. Congestion of the mucous membrane* itself; a rose or cherry colored, (never livid or violet,) redness, (rosige oder kirschrothe roethe,) which occupies large spaces of the mucous membrane, which, at times, extends to the size of 1 to 2″, especially in the smalli ntestines; at others, and oftener, it is confined to small spots or folds; and, at other times, again, it has the form of the aborizing branch of a blood-vessel. This redness extends, also, over the normal tissue of Peyer's patches, with the congestion, the adhesion of the mucous membrane with the serous, is very much diminished, but the consistence of the former is normal. The isolated glands are not visible as is the case in health.* The congestion exists alone or in connection with inflammation of other parts of the mucous membrane, or that of its glands. This congestion is obviously a morbid increase, (Steigerung,) of the physiological condition; the diminished adhesion is in consequence of the increased amount of fluid in the submucous cellular tissue.

*B. Congestion of Peyer's patches.*—These are more easily visible, partly on account of their more areolar structure, partly on account of their partial (confined to isolated spots on the same) redness, whilst the rest of the mucous membrane is of the natural color. At the same time, the isolated glands, in the small and large intestines, are plainer to be seen, without, however, overreaching the level of the mucous membrane. This change, is certainly one of the most frequent causes of the transitory diarrhœa, which lasts but a short time, and occurs so frequently, even in strong children, especially during the process of teething.

---

*This seems to contradict the description of the normal condition of the glands.

*Query.*—Wherein consists the difference, (if there is any,) between Lieberkuen's and the isolated glands?—Translator.

B. ACUTE INFLAMMATION.

*A.* It occurs in *acute erythematic inflammation*, but the authors have seen it, only, occupying small surfaces, and accompanied by severe diseases of other parts connected with the mucous membrane. Nor has it ever occurred in any considerable degree, in the isolated glands; but always either as a subordinate affection, with other severe diseases, or as an ulcerative inflammation.

*B. Primary acute inflammation of Peyer's glands*, is one of the most important, and not a rare disease of infancy. A more or less considerable number of the patches (generally ten or twelve, never all,) and not exclusively near the coecum, show a remarkable even cherry colored redness, and considerable puffing, and thereby a prominence above the mucous membrane. The redness is, on some, equally extended; on others, especially on those in which the disease has just commenced, in spots, here and there, occupying only the edges. The puffing out is a constant symptom, and shows itself from the single bold-relief to the genuine granulated appearance. In the latter case, the patch has the appearance as if it were laid in with innumerable small red grains. The consistence, especially of those most puffed up, is diminished, and the tissue, after an incision, is easily scraped off with the back of a knife. The redness, puffiness and diminished consistence comcommence always, gradually, at first confined to small spots of single patches; then come generally, diseaed patches closer together, but between two diseased ones, are found such as are either sound, or only congestive. Often the patches thus deceased, are visible through the serous membrane, from without, and sometimes such places are indicated by great injection of the latter membrane. The Peyer patches thus reddened, and puffed, contrast greatly with the surrounding normal or slightly erythematic muc. mem. The isolated glands are either normal, or there are but few diseased. The mesentoric glands are mostly enlarged, a little injected, but of firmer consistence.

*C. Secondary acute inflammation of Peyer's glands.*—The authors have found this inflammation only with tubercles; analogous to it, is the inflammation of the same organ in eruptive fevers, especially in scarlet. Its peculiarity consists in the circumstance that in consequence of its dependance on a disease of the skin, the inflammation does not appear so pure, and is more easily complicated, than in the primary form. Besides, in another principal affection, more or less numerous glandular plexus, manifest themselves, either uniformly, or in separate patches, deeply red, puffed, and in places even ulcerated. Also ulcerative inflammation of the isolated follicles of the small intestine, and simple development of the isolated glands of the large.

C. CHRONIC INFLAMMATION OF PEYER'S GLANDS.

This is the most frequent cause of the atrophy and marasmus, (abzehrung,) of the infant. Its anatomical character, is as follows: several glandular tissues show a coloring, which is, sometimes, entirely gray-blue, and sometimes, more slate-gray; and this contrasts much with the other, mostly pale mucous membrane, which latter, however, can, in but rare instances, in consequence of chronic inflammation, in places, be colored livid-gray. These deceased patches are much more visible than the healthy ones. Their areolar structure is effaced, the edges are sharply limited, with black points thereon. Sometimes their tissue is very thin, at others, there is some puffiness, according to the stage in which death takes place. *This chronic inflammation of Peyer's glands*, exists either, (and mostly,) alone, or there are, also, as a local complication, traces of inflammation in the mucous membrane, or in the isolated glands. The mesenteric glands, are never essentially changed. There are never complications with tubercles; but there is a great disposition to complication with other organs. (Pneumonia lobularis, hydrocephalus, &c., &c.)

D. EXULCERATION OF THE ISOLATED GLANDS.

*A. Primary exulceration.*—The proper primary inflammatory swelling, and exulceration of the isolated glands, is, upon the whole, a proportionably rare occurrence, whilst a swelling of the same, without inflammation, (so that they appear upon the mucous membrane, as small, whitish bodies, of the size of a pin-head, or millet-seed,) takes place frequently in all other affections of the intestinal mucous membrane and its annexæ. The ulcerative inflammation of the isolated glands, constitutes, like the chronic inflammation of Peyer's glands, but more rarely the anatomical foundations of atrophy. Its characteristics are the following: a more or less extensive surface of the mucous membrane of the ilium is coated with small round ulcers of the size of a millet-seed, or lentil, answering to that of the isolated glands. They are, sometimes, isolated and scattered, at others, in patches, or two or three run into each other. They are not perceptible on the outside of the gut. There are no tubercles either on the ulcerated surface, nor on any other part. The isolated glands of the colon, Peyer's and the mesentric glands, remain either healthy, or are not essentially effected.

*B. Secondary exulceration.*—This is, in part, a manifestation of tubercles, and appears as such, frequently, with the secondary inflammation of Peyer's glands. The morbid changes of the isolated and Peyer's glands, in this case, resembles much, those which we, under similar circumstances, find in tuberculosis adultorum ; yet in the latter there is often a perceptible deposition of tubercular substance at the bottom of the ulcer, and externally on the gut, which in children, is not the case. The anatomical characters are the same as in the primary exulceration, with this difference, that the exulcerated glands are surrounded by a strong, inflammatory halo. At the same time disease of Peyer's glands appear always to take place, and both are dependant on the presence of tubercular affections.

E. SOFTENING OF THE INTESTINAL MUCOUS MEMBRANE.

This is a frequent post-mortem appearance, and its character is of that kind, as not to admit of doubts, such as have been raised about the softening of the stomach, we can distinguish two varities.

*A. Red softening of the intestinal mucous membrane:* a space of the mucous membrane, mostly of the small intestines, of more or less extent, has lost its consistence in such a degree, that it can easily be scraped off with the back of a knife. Its color is on its larger surface normal, only on a few small, but connected spots, is seen a rosy tint which cannot be scraped off; there is, withal, a diminished adhesion even in places where there is no decrease of consistence. The tissure of the mucous membrane is frequently infiltrated, as if with serum, or as if chahged into a homogenous jelly. In such places the texture of the mucous membrane cannot be recognized. The walls of the intestinal canal are, in such places, easily torn. The changes which at the same time take place in the isolated glands, Peyer's patches and messenterial glands, are of inferior importance.

*B. White softening of the intestinal mucous membrane.*—The anatomical character is the same as in the red softening, only the mucous membrane, (like the whole wall of the gut,) is distinguished by a remarkable paleness according as the muc. mem. is simply softened, or more gelatinous, (more succulent) do we find the mass, removed with the back of a knife, and which is the residue of the mucous membrane, more or less voluminous. Here, also, is always diminished adhesion. This white softening occupies larger spaces than the red, obviously, in consequence of its longer existence. Peyer's patches are, without any other change, more easily perceptible, probably, because of the greater paleness of the mucous membrane. This white softening, always of a chronic character is, also, one of the post-mortem appearances in cases of atrophy in children. The gradual transition of the red, into the white softening, and the analogous appearances in other organs (e. g. in the brain) proves, that the starting point of this white softening is an inflammatory condition of the tissue of the mucous membrane.

The authors now think themselves justified in the following *resumé:* 1. The changes of the intestinal mucous membrane are frequent, perhaps the most frequent, post-mortem appearances of the infant age. 2. These changes are partly the chronic, (the principal foundation of atrophy,) partly the acute (the conditional causes of the acute, very exhausting diarrhœa, which is often accompanied by cerebral affections and of many diagnosticated diseases, as softening of stomach. 3. These changes are in the same proportion frequent post-mortem appearances in the above mentioned conditions, as the most of those generally enumerated, are rare (such as enlargement of the mesenteric glands, softening of the stomach, aphthæ, &c., &c.) 4. These changes, likewise, are much more frequent than those of the stomach, which latter are, with the exception of the softening of the cul-de-sac, comparatively rare in infancy. 5. The changes in the mucous membrane, except the secondary ones, are in cadavers mostly separate. 6. The most frequent of all changes, is the chronic inflammation of Peyer's glands; this is also the most frequent anatomical foundation of the atrophy of children. 7. Next to this, but not more rarely, occurs the red and white softening as the cause of atrophy. 8. These softenings are but different stages of the same process; the simple and gelatinous softening, but different forms. 9. A more rare post-mortem appearance is the chronic exulceration of the isolated glands of the small intestines. 10. A disease, as yet, never appreciated, and very dangerous, is the actute inflammation of Peyer's glands. 11. This disease is a true phlogosis, as is also evident from its concomitant diseases, (croup, pneumonia.) 12. Most of authors don't know this disease, and the few who have seen its changes, confound it with dothinentoritis, which does not occur in the first year of life. 13. The secondary acutte inflammation of Peyer's glands and inflammation of the isolated glands, which exist mostly at the same time, form part of the tuberculous disease. The authors have always, in this case, found tubercles in the spleen, never in the intestines. 14. The authors have often observed the colitis of the French, but always con fined to small spaces, and obviously as a slight affection in comparison to the other changes in the mucous membrane of the small intestine, which existed at the same time. 15. The mesenteric glands show themselves, except trifling redness and enlargement, in some cases, in a normal condition, their affection is never severe. Only in a general tubercular affection are they often partially infiltrated with tuberculous deposition. 16. The peculiarity and frequency of the above mentioned changes of the intestinal mucous membrane, on the one hand, and the rare occurrence of many diseases, already so important, in the second year (typhus, intestinal, tubercles) on the other, form one of the most prominent characteristics of the pathology of the intestinal mucous membraue in the age of infancy.

Remark of the translator.—In order to transmit accurately the anatomical and pathological descriptions, I have preferred, in many places, a *verbal translation*, if it would convey the authors meaning plainly, to a *smoother style*, that might give room to ambiguity. I hope this remark will be received, in part at least, as an apology for the stiffness, &c., &c., of the style of this paper.

II.

*Bonnet*, Chirurg. des Hôtel-Dieu at Lyon, communicates in the Gazette de Paris, numbers 15, 16, and 18, 1843, in a long article, his experience and observations in relation to *cauterization as a preventive of phlebitis and of the absorption of pus*. He says, 1st. That superficial veins can be opened and destroyed with kali caustic, caustic vien, and zinc muriat., without producing supporative phlebitis. 2d. Has phlebitis occurred in consequence of a simple or poisoned wound, then is cauterization with the hot iron a powerful remedy to stay the phlebitis in its progress. 3d and 4th. Hemorrhoidal, and other tumors, can also be safely destroyed by cauterization. 5th. The destruction of the whole inner surface of large abscesses with a hot iron or zinc. mer. prevents all bad consequences, which may follow the opening of large abscesses; cauterization can even stay these consequences.

